# Depolarized Forward Light Scattering for Subnanometer Precision in Biomolecular Layer Analysis on Gold Nanorods

**DOI:** 10.1021/acs.jpclett.4c02956

**Published:** 2025-01-27

**Authors:** Peter Johansson, Mikael Käll, Hana Šípová-Jungová

**Affiliations:** † School of Science and Technology, Örebro University, 701 82 Örebro, Sweden; ‡ Department of Physics, Chalmers University of Technology, 412 96 Göteborg, Sweden

## Abstract

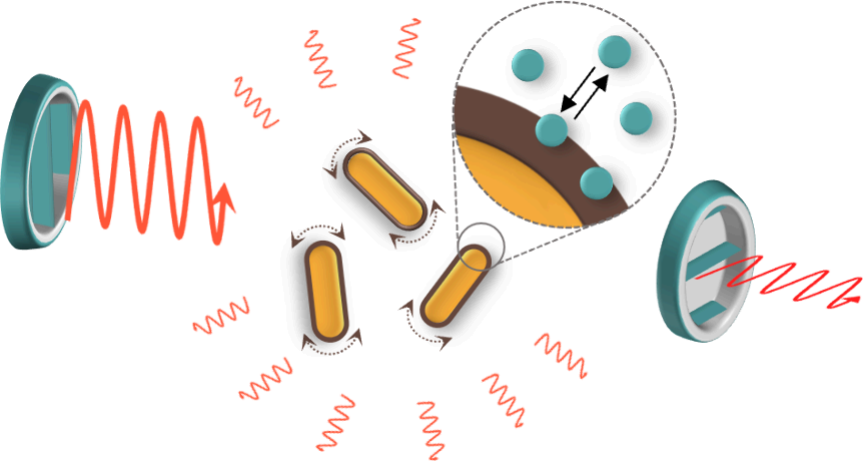

Functional gold nanoparticles have emerged as a cornerstone in targeted drug delivery, imaging, and biosensing. Their stability, distribution, and overall performance in biological systems are largely determined by their interactions with molecules in biological fluids as well as the biomolecular layers they acquire in complex environments. However, real-time tracking of how biomolecules attach to colloidal nanoparticles, a critical aspect for optimizing nanoparticle function, has proven to be experimentally challenging. To address this issue, we present a depolarized forward light scattering (DFLS) method that measures rotational relaxation constants. In DFLS, optically anisotropic nanoparticles are illuminated with linearly polarized light and the forward light scattering is analyzed in a cross-polarized configuration. We demonstrate the application of DFLS to characterize various functional coatings, analyze biomolecular binding kinetics to gold nanoparticles, and determine specific protein adsorption affinity constants. Our results indicate that DFLS offers a powerful approach to studying nanoparticle-biomolecule interactions in complex environments such as bodily fluids, thereby opening new pathways for advancements in nanomedicine and the optimization of nanoparticle-based drug delivery systems.

In recent decades, nanoparticles (NPs) have emerged as pivotal tools in nanomedicine, serving as carriers for imaging agents, therapeutic molecules, and transporters delivering biological materials to specific sites in the human body.^1,2^ The functionality of NPs in living systems relies heavily on their interactions with the surrounding environment, including proteins, cells, and tissues.^3,4^ Within biological environments, NPs typically develop a “corona” of adsorbed molecules,^5,6^ which greatly impacts their behavior and fate within the body.^7,8^ It is therefore crucial to have methods that can characterize and quantify the interactions between NPs and biomolecules in order to advance from laboratory research to real-world applications in nanomedicine. However, characterizing the kinetics of nanoparticle interactions in real time has been a significant challenge.^9^

Although the morphology of NPs can be examined with subnanometer resolutions using microscopy techniques like electron microscopy,^10^ scanning probe microscopy,^11^ and through X-ray diffraction,^12^ these methods predominantly operate under high-vacuum, dry, or surface-immobilized conditions. Consequently, they are ill-suited to explore critical phenomena occurring in suspension, such as particle swelling or dynamic biomolecular interactions. Traditional analyses of colloidal suspensions often resort to techniques rooted in light scattering, diffusion, and sedimentation, which yield insights into the particle size, distribution, concentration, and aggregation states. The most commonly used method is dynamic light scattering (DLS),^13^ in which temporal fluctuations in intensity of light scattered by particles in suspension are used to calculate particle hydrodynamic diameters. However, DLS requires well-controlled experimental conditions to ensure accuracy, and struggles with resolving subnanometer variations in hydrodynamic diameter.^14,15^ An alternative approach is nanoparticle tracking analysis (NTA), which determines particle size by examining the free diffusion behavior of NPs in solution.^16^ NTA excels in analyzing polydisperse samples but falls short in evaluating the biomolecular interaction kinetics. Techniques like fluorescence correlation spectroscopy (FCS) and fluorescence cross-correlation spectroscopy (FCCS) can offer valuable insights into biomolecular binding^17^ but rely on labeling and is vulnerable to photobleaching.

Depolarized dynamic light scattering (DDLS) has recently emerged as a noninvasive alternative method for exploring the rotational Brownian motion of optically anisotropic nanoparticles in liquid media.^18,19^ This technique represents an evolution of traditional DLS in that it utilizes linearly polarized light for illumination and analyzes cross-polarized and copolarized scattered light from colloidal suspensions to determine rotational and translational diffusion coefficients of dispersed nanoparticles.^18^ DDLS’s broad applicability has enabled the characterization of a wide array of nanoparticles, including cellulose nanocrystals,^20^ tobacco mosaic viruses,^21^ carbon nanotubes^22^ and silver nanoplatelets.^23^ Several recent studies have focused on gold nanorods due to their importance in many nanomedicine applications.^24,25^ Gold nanorods are suitable for DDLS investigations due to the localized surface plasmon resonance (LSPR) phenomenon, which greatly enhances the intensity of depolarized light scattering.^26^ This allows for the extraction of translational and rotational diffusion constants,^27,28^ nanorod dimensions^18^ and shape details,^29^ and the detection of impurities.^30^ DDLS has also been successfully used to investigate functional layers like polyethylene glycol (PEG) coatings on gold nanoparticles^31^ and carbon nanotubes,^22^ as well as temperature transitions of thermoresponsive polymers.^32^ However, previous implementations of DDLS typically involved measurements over a range of scattering angles to assess both translational and rotational diffusion properties. This approach is technically demanding and time-consuming and, therefore, presents challenges in reaching the time resolution needed for fast interaction kinetics.

Here we present an enhanced and streamlined variant of DDLS for real-time, in situ studies of biomolecular interactions on suspended gold nanorods. Unlike previous methodologies, our approach employs DDLS in a transmission configuration, which effectively isolates the contribution of rotational diffusion to the DDLS signal by eliminating the effects of translational Brownian motion, thereby enhancing the data acquisition speed. We term the method depolarized forward light scattering (DFLS). Importantly, the intensity of the depolarized scattering is boosted in the DFLS configuration since the gold nanorods in the relevant size range (∼100 nm) exhibit superior forward scattering amplitude, relative to the traditional 90° scattering geometry.^33^ Additionally, by performing DFLS measurements at wavelengths near the longitudinal LSPR of the nanorods (here, around 785 nm), we achieve a substantial signal increase. This enhancement allows us to detect small changes in nanorod dimensions and to determine the thicknesses of model biomolecular layers with subnanometer resolution. As a proof-of-principle application, we use DFLS to determine thicknesses of model biomolecular layers composed of alkanethiols and polyethylene glycol (PEG). PEG is often selected for surface modification of therapeutical NPs due to its advantageous characteristics, including electrical neutrality, significant spatial repulsion, and high hydrophilicity.^34^ We study the kinetics of PEG binding to the nanoparticle surface and nonspecific adsorption of model protein to grafted PEG, and monitor conformational changes of PEG in solutions of various ionic strengths. These applications demonstrate the potential of the method for dynamic studies of biomolecular interactions and protein corona formation.

## DFLS Instrumentation and Nanorod Characterization

**Figure 1:**
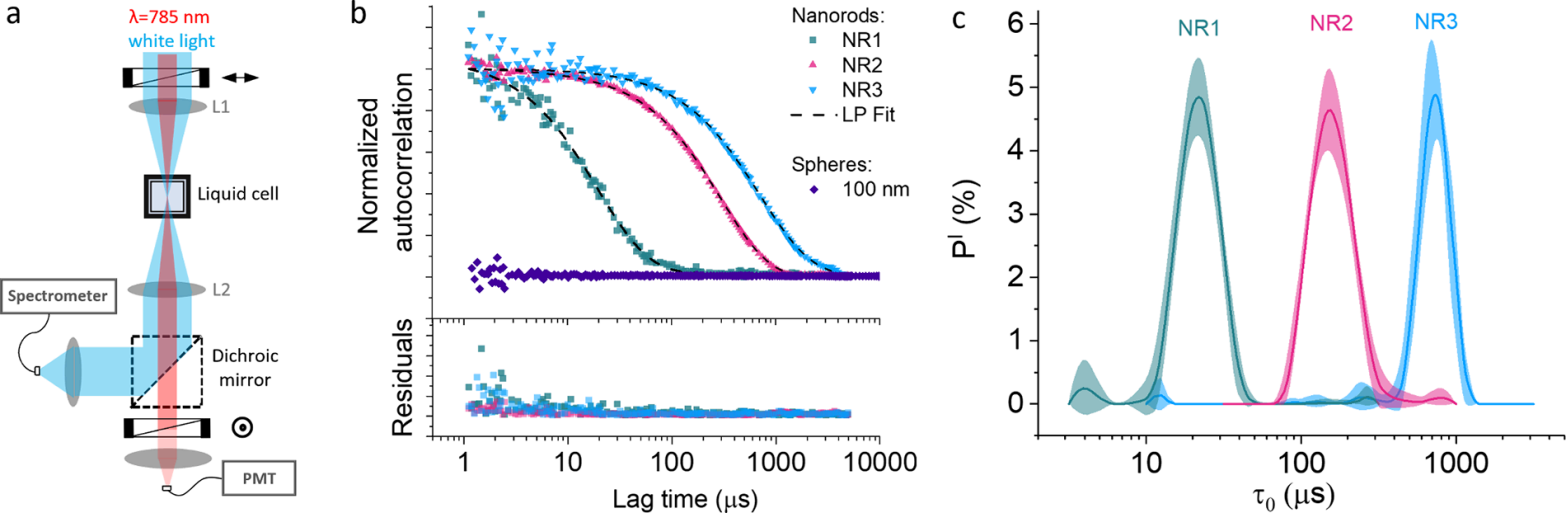
DFLS setup and characterization of nanoparticle size distributions. (a) Schematic of the experimental setup used for simultaneous measurements of DFLS and extinction spectroscopy in transmission mode. (b) Top: Normalized depolarized ACFs of three different sizes of nanorods and spherical gold nanoparticles with diameter 100 nm. Dashed lines show inverse-Laplace curve fits. Bottom: Residuals of the fits. (c) Normalized probability distributions *P*^*I*^(τ_0_) of the ACF decay times derived from the fits shown in (b); the lines represent averages from 10 measurements. The peak positions of the distributions, obtained from Gaussian fits to the data points, are NR1: 29 ± 0.6 μs, NR2: 255 ± 6 μs, and NR3: 845 ± 25 μs.

Figure 1a provides an overview of the custom-built instrument used for depolarized forward light scattering. Briefly, the setup involves a single-mode laser emitting at a wavelength of 785 nm, which is collimated, linearly polarized, and weakly focused into a cuvette containing a solution of gold nanorods (NRs). The light scattered in the forward direction passes through a second polarizer set at a cross-polarized angle before being collected by a photomultiplier tube (PMT) connected to a digital autocorrelator. The setup also includes extinction spectroscopy for real-time monitoring of localized surface plasmon resonance (LSPR) shifts, providing additional information about biomolecular binding and potential nanoparticle aggregation (Figure 1a).

To demonstrate the instrument’s capabilities, we performed DFLS measurements on nanorod solutions of three different sizes, synthesized via the seeded-mediated growth method.^35,36^ The nanorod samples had approximate dimensions (length × width) of 65 × 21 nm^2^ (NR1), 140 × 72 nm^2^ (NR2), and 190 × 127 nm^2^ (NR3), with concentrations of 1 × 10^10^ particles per mL (see Supporting Information (SI), Figure S2 and Table T1, for SEM images and details on size polydispersity). As shown in Figure 1b, the scattering autocorrelation functions (ACFs) of the nanorod solutions display decaying behavior, with the autocorrelation decay time τ_0_ increasing as the nanorod size increases. As expected, a solution of 100 nm diameter gold spheres exhibited no discernible DFLS signal, confirming the method’s specificity for anisotropic particles. The DFLS measurements of nanorod solutions yielded high signal-to-noise ratios, despite using a relatively modest laser power of 10 mW and a short integration time of 5 s per ACF. This efficiency makes the technique suitable for high-temporal-resolution measurements of nanorod–biomolecule interaction kinetics, which we discuss further below.

As we demonstrated previously,^37^ the autocorrelation decay time τ_0_ can be related to the geometrical parameters of a nanorod through the following equation:
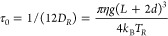
1where *D*_*R*_ is the rotational diffusion constant, η is the temperature-dependent dynamic viscosity of the medium,^38^*L* is the length of the nanorod, *d* is the thickness of an adsorbed biomolecular layer, and *g* is a geometrical factor that depends on the nanorod excentricity.^37,39^ The rotational Brownian temperature *T*_*R*_ equals the ambient temperature *T*_amb_ in the absence of photothermal effects. However, due to localized surface plasmon resonance (LSPR) excitation, which enhances light absorption and photothermal heating, *T*_*R*_ may exceed *T*_amb_.

Even with the relatively low polydispersity of the nanorod solutions used in this study (see SI, Table T1), minor variations in size led to the ACF displaying a range of decay times. These decay times are represented by a probability distribution, *P*^*I*^ (τ_0_), which can be derived from the ACF using an inverse Laplace transformation. However, this mathematical process is inherently ill-posed, often resulting in multiple possible solutions, unless additional constraints are applied.

To address this, we employed the constrained regularization method, CONTIN,^40,41^ recently adapted into MATLAB by Marino.^42^ Our approach incorporated the L-curve criterion^43,44^ as a regularization strategy, a method that has been shown to provide more reliable results than the original CONTIN implementation.^45^ The derived *P*^*I*^ (τ_0_) distributions for NR1, NR2, and NR3 are shown in Figure 1c. The resulting fits to the ACF functions align well with our experimental data, as seen in the upper section of Figure 1b, with the residuals displayed in the bottom panel, confirming the accuracy of the fits.

## Intensity- and Size-Weighted DFLS Distributions

**Figure 2:**
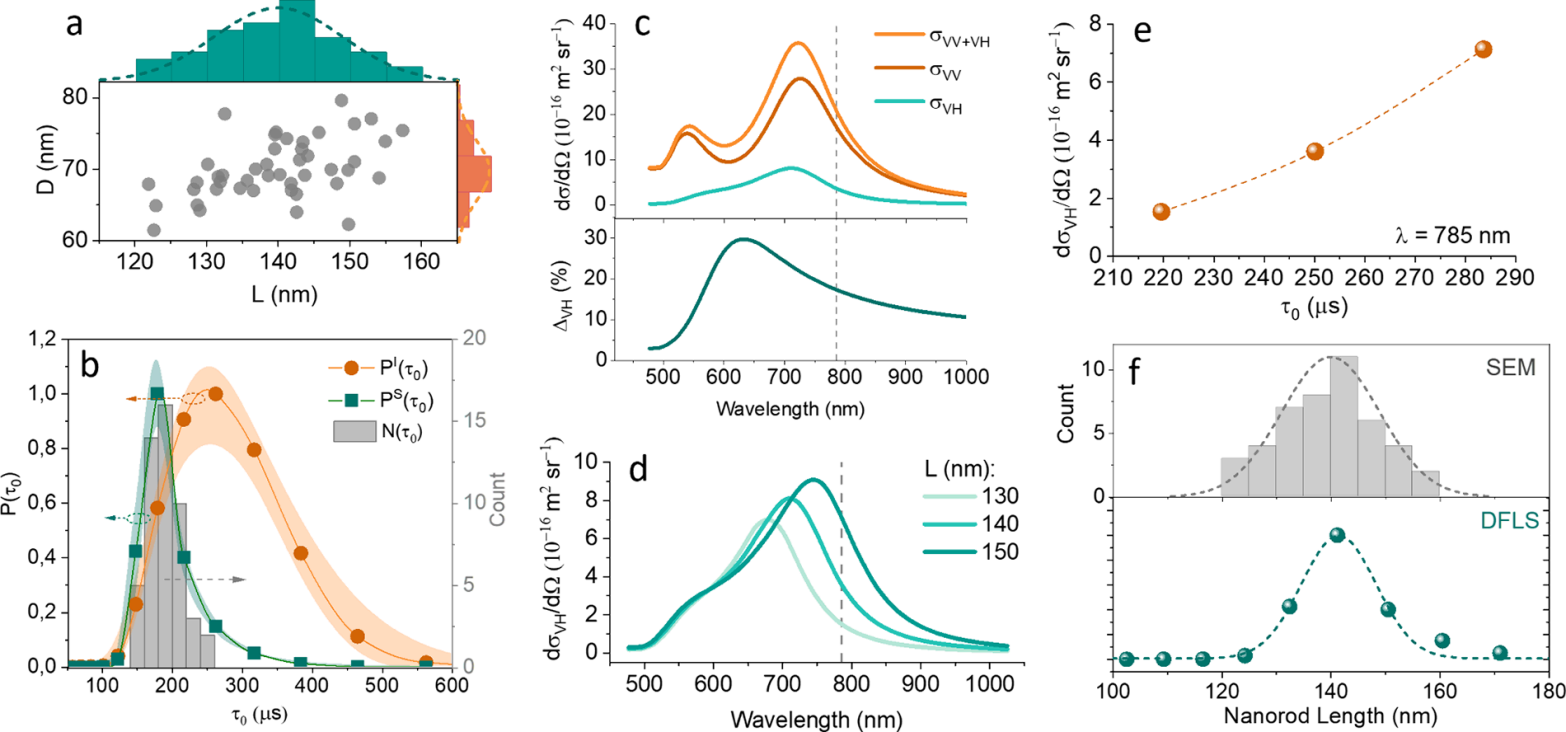
Size characterization of nanorods (NR2) using SEM and DFLS measurements. (a) SEM analysis of 40 nanoparticles, showing their length (*L*) and width (*D*), with average values being *L* = 140 ± 9 nm and *D* = 70 ± 4 nm. (b) DFLS data averaged over 20 measurements presented as *P^I^*(τ_0_). *P^S^*(τ_0_) represents the size distribution calculated using eq 2. Gray bars show τ_0_ distribution calculated using eq 1 based on individual nanorod dimensions determined via SEM measurements. (c) Forward scattering cross sections of NR2 based on T-matrix simulations, including total (*σ*_VV_ + *σ*_VH_), copolarized, (*σ*_VV_ and cross-polarized (*σ*_VH_) cross sections and depolarized ratio Δ_VH_ as a function of wavelength. Laser wavelength is indicated with dashed line. (d) Variation in the depolarized scattering cross-section with nanorod length, with a fixed width of 70 nm and lengths of 130, 140, and 150 nm. (e) Dependence of *σ*_VH_ on τ_0_ at laser wavelength of 785 nm and ambient temperature of 293 K. The dashed line represents a fit with a second-order polynomial. (f) Comparison of length distributions obtained from SEM and DFLS (*P^S^*(τ_0_)) showing strong agreement between the two methods.

We aimed to determine whether the intensity probability distribution *P*^*I*^(τ_0_) obtained with DFLS aligns with the size-based probability distribution *P*^*S*^(τ_0_) derived from actual measurements of the nanoparticle sizes in the sample. To do this, we first measured the dimensions of 40 NR2 nanorods by using SEM (Figure 2a), finding an average width of 140 ± 9 nm and an average length of 70 ± 4 nm. Using eq 1, we calculated the characteristic times τ_0_ for each nanorod, including an additional 4 nm on both the width and length to account for the CTAB double-layer (shown as bars in Figure 2b). Next, we compared the calculated τ_0_ distribution from SEM data with the *P*^*I*^(τ_0_) measured with DFLS (represented by dots on Figure 2b). The comparison revealed the *P*^*I*^(τ_0_) distribution is shifted toward larger values, indicating a discrepancy between the DFLS measurements and the SEM-derived size calculations.

This discrepancy indicates that larger nanorods contribute disproportionately to the DFLS signal compared with smaller ones. To investigate this further, we examined the relationship between the forward depolarized scattering cross-section (σ_VH_) and nanorod size using T-matrix calculations (see Supporting Information for more details). Figure 2c shows the calculated total (σ_VV_+ σ_VH_), copolarized (σ_VV_) and cross-polarized (σ_VH_) cross sections of forward scattering at different illumination wavelengths for a nanorod of size of 140 nm × 70 nm. The depolarized ratio defined as Δ_VH_ (%) = 100 × σ_VH_/(σ_VV_ + σ_VH_) peaks at 30% at a wavelength of 630 nm and decreases to 19% at 785 nm (Figure 2c, bottom).

In Figure 2d, we explore how σ_VH_ varies with nanorod length while fixing the width at 70 nm. We tested nanorods with lengths of 130, 140, and 150 nm. We observed that increasing the length shifts the peak λ_VH_^max^ to longer wavelengths and increases the scattering intensity at 785 nm (Figure 2d). This confirms the hypothesis that larger rods contribute disproportionately to *P*^*I*^(τ_0_), influencing the intensity-weighted distribution more than their actual abundance in the sample would suggest.

To account for this effect, we used the relationship between the nanorod size and scattering intensity to correct the size distribution *P*^*S*^(τ_0_). The corrected size distribution is calculated as
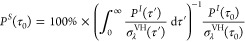
2where σ_VH_ (τ_0_) represents the empirically derived relationship between the scattering cross-section and τ_0_ obtained from T-matrix calculations (Figure 2e). The normalization factor in parentheses ensures that the total probability of the size distribution sums to 100%.

Finally, Figure 2f compares the corrected size-based distribution *P*^*S*^(τ_0_) with the intensity-weighted distribution *P*^*I*^(τ_0_) for NR2 at 785 nm. We found that the size distribution *P*^*S*^(τ_0_) aligns well with the τ_0_ distribution derived from SEM measurements, validating that the primary difference between the size-based and intensity-weighted distributions arises from the variation in scattering intensity with particle size. This difference can be accurately corrected by using the relationship in eq 2.

## Temperature Sensitivity and Contribution of Plasmonic Heating

When illuminated at their plasmonic resonance wavelength, gold nanoparticles efficiently absorb light and can, therefore, serve as nanoscale heat sources. To explore how LSPR-enhanced light absorption affects the DFLS signal, we analyzed the decay rates across a range of nanoparticle concentrations 0.5–5 × 10^10^ particles/mL; SI, Figure S4). As shown in SI, Figure S4b, increasing nanoparticle concentration leads to broadening of the *P*^*S*^(τ_0_) distribution and shift of its maximum toward lower values. This shift likely results from an increase in solution temperature, which decreases the solution’s viscosity due to localized plasmonic heating. Additionally, the broadening of the distribution may be attributed to multiple scattering events, where a photon is consecutively scattered by several particles. These findings underscore the importance of carefully controlling the nanoparticle concentration, which should ideally remain below 5 × 10^10^ nanoparticles/ml for nanoparticles with LSPR overlapping with the excitation wavelength. In subsequent experiments, we used a concentration of ∼1 × 10^10^ nanoparticles/ml. At lower concentrations, the DFLS signal becomes excessively noisy and the extinction spectra of the solution are difficult to distinguish (SI, Figure S4a).

We further investigated the heating effect at this low nanoparticle concentration by adjusting the laser power. As anticipated, the amount of light absorbed, and consequently the temperature increase, was proportional to the laser power. As we increased the laser power from 12.5 to 45 mW, we observed a broadening in the *P*^*S*^(τ_0_) decay time distribution and a decrease in the decay time maximum (τ_0_^max^; SI, Figure S5). To quantify the localized heating effect influencing rotational Brownian motion, we calculated the local temperature *T_R_* using eq 1. The results show a modest temperature rise of 1.5 K at 12.5 mW and 4.5 K at 45 mW compared to ambient temperature, i.e., relatively small temperature increase for low laser powers.

## Temperature-Dependent Transitions in CTAB Layer Thickness

**Figure 3:**
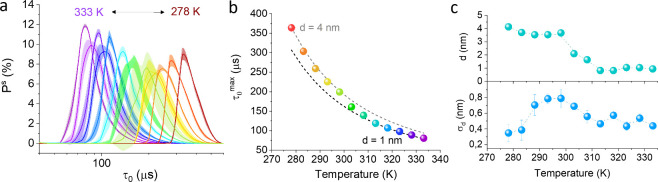
Effect of temperature on CTAB double-layer on NR2 nanorods. (a) Decay time distribution at various bulk temperatures averaged from 10 measurements calculated using eq 2 and σ_VH_ (τ_0_) shown in SI, Figure S7. (b) Distribution maxima (τ_0_^max^) as a function of the temperature. Dashed lines represent theoretical values calculated using eq 1. for *L* = 139 nm and biomolecular layers of 4 and 1 nm, respectively. (c) Calculated biomolecular layer thickness (top) and distribution width (bottom) as a function of temperature. The results suggest CTAB detachment from nanorods at elevated temperatures.

We now turn to applying the DFLS technique to the characterization of various biomolecular layers on the nanorods. To investigate how variations in bulk solution temperature influence the CTAB double-layer that stabilizes NR2 nanoparticles in solution, we employed a Peltier-controlled liquid cell to modulate the temperature between 280 and 335 K. This temperature modulation led to a significant shift in the *P^S^*(τ_0_) decay time distribution, with distribution maxima τ_0_^max^ shifting from 364 ± 2 μs at 280 K to 80 ± 1 μs at 333 K, as shown in Figure 3a,b. In Figure 3b, we plotted the distribution maxima against temperature, alongside theoretical τ_0_^max^ values derived from eq 1, which incorporates the temperature-dependent viscosity of water and the friction coefficient for a nanorod with biomolecular layer thicknesses of 3 and 1 nm, respectively. The results show a decrease in the thickness of the CTAB layer with increasing temperature, with a transition occurring around 300 K.

Using a calibration curve *d*(τ_0_^max^) for each solution temperature and viscosity (example is shown in SI, Figure S8), we determined the average thicknesses of the biomolecular layers on the NR2 surface at different temperatures (Figure 3c). Our results indicate that while the CTAB double-layer remains intact on the nanorods below 290 K, partial dissociation occurs above 300 K. This behavior is reflected in the distribution width, which peaks around 300 K, indicating an increased heterogeneity in the CTAB layer. Notably, the distribution width begins to expand at 290 K, suggesting that the disorganized state of the double-layer precedes dissociation at higher temperatures. As the CTAB double-layer dissociates at temperatures above 310 K, the distribution narrows once again. To assess the reversibility of this transition, the temperature was gradually increased from 298 to 333 K and then returned to 298 K. The decay times measured at 298 K before and after the temperature cycle were nearly identical (SI, Figure S6), indicating that the transition is fully reversible. These findings demonstrate that DFLS is a sensitive tool for detecting subtle changes in nanoparticle size caused by temperature-dependent transitions.

**Figure 4:**
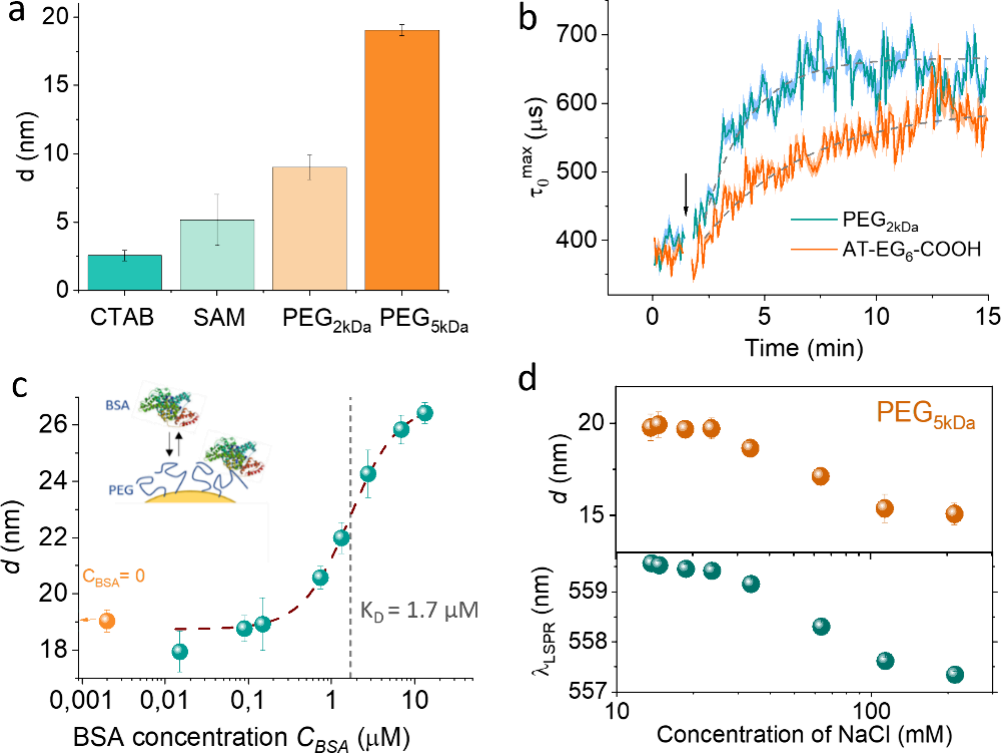
Analysis of biomolecular interactions on nanorods using DFLS. (a) Biolayer thicknesses of various coatings on NR2 nanorods, including CTAB double layer, 11-MU-EG_6_-AA self-assembled monolayer (SAM), and PEG layers with molecular weights of 2 kDa and 5 kDa (average of five 1 s measurements) (b) Time-resolved changes in τ_0_^max^ showing the kinetics of PEG_2kDa_ and 11-MU-EG_6_-AA adsorption onto NR2 nanorods. The arrow indicates the introduction points of PEG_2kDa_ and 11-MU-EG_6_-AA. (c) Biolayer thickness of NR2 functionalized with PEG_5kDa_, plotted against varying BSA concentrations in a 10× diluted PBS solution. The logistic regression curve (dashed line) is utilized to determine the BSA-PEG_5kDa_ interaction’s dissociation constant (*K*_D_). (d) Analysis of PEG_5kDa_ layer thickness on NR2 within a 10× diluted PBS buffer as a function of increasing NaCl concentrations. The top panel displays DFLS measurements of PEG layer thickness, while the bottom panel correlates these findings with shifts in the transversal LSPR spectral position.

## PEG and BSA Adsorption Kinetics and Layer Behavior under Varying Ionic Strengths

We quantified the thicknesses of various functional coatings on NR2 nanoparticles, including self-assembled monolayers (SAMs) of alkanethiols (11-MU-EG_6_-AA) and polyethylene glycol (PEG) coatings with molecular weights of 2 and 5 kDa, as shown in Figure 4a. The CTAB coating thickness was measured to be 2.6 ± 0.4 nm, the SAM was 5.2 ± 1.9 nm, and the PEG layers were 9 ± 0.9 nm and 19 ± 0.4 nm, respectively. These results are consistent with our previous research^37^ and with literature values.^46^ Remarkably, the DFLS technique achieved subnanometer accuracy in determining the thickness of biomolecular layers.

To further demonstrate DFLS’s capability to study biomolecular interactions in real time, we investigated the adsorption kinetics of PEG_2kDa_ and 11-MU-EG_6_-COOH onto CTAB-coated nanorods (Figure 4b). Initially, the DFLS signal of CTAB-coated NR2 was monitored over several minutes to establish a baseline. Upon injection of the functional molecules, their binding induced a gradual increase in the hydrodynamic radius of the nanorods. Notably, the adsorption rates differed between the two biomolecular coatings, highlighting the potential of the DFLS to capture real-time adsorption kinetics.

Additionally, we studied the adsorption behavior of bovine serum albumin (BSA), a commonly used protein model, onto PEG_5kDa_-coated nanorods (Figure 4c). In a 10× diluted PBS buffer, the PEG_5kDa_ layer exhibited a thickness of 18.9 ± 0.2 nm. We then gradually increased the BSA concentration by injecting a concentrated BSA solution into the cuvette containing the nanorods, measuring the DFLS signal five min after each injection. The results, shown in Figure 4c, illustrate the biolayer thickness as a function of BSA concentration. Data were fitted with a sigmoidal function *d* = *d*^max^/(1 + exp(−*k*(*C* – *K*_D_))), where *d*^max^ represents the maximum layer thickness at high BSA concentrations, *k* is the growth rate, *C* is the bulk BSA concentration, and *K*_D_ is the dissociation constant for the interaction between PEG_5kDa_ and BSA, yielding *d*^max^ = 26.4 ± 0.2 nm. After subtracting the thickness of the PEG_5kDa_ layer, this suggests a BSA layer thickness of approximately 7.4 ± 0.6 nm, corresponding to near-monolayer coverage at high BSA concentrations. The equilibrium affinity constant, *K*_D_, was determined to be 1.7 ± 0.2 μM, in reasonable agreement with a *K*_D_ of 1.4 ± 0.1 μM obtained through parallel measurements of the transverse LSPR resonance (SI, Figure S8).

Lastly, we examined the effect of the NaCl concentration on the thickness of PEG_5kDa_ layers in diluted PBS solutions (Figure 4d). Increasing the NaCl concentration resulted in a significant reduction in PEG_5kDa_ layer thickness, from approximately 20 nm at low salt concentrations to 14 nm at 100 mM NaCl and above. To determine the cause of this reduction, we combined the DFLS measurements with LSPR tracking. The shift in LSPR spectral position at higher ionic strengths suggested a loss of mass, indicating the dissociation of noncovalently bound PEG molecules in high-ionic-strength environments. This finding underscores the utility of combining DFLS and LSPR tracking for studying biomolecular adsorption phenomena such as polymer swelling or dissociation.

For future optimization of the DFLS setup, we note the potential benefit of implementing a narrow-band filter to block only the laser wavelength while preserving the detection of the remaining spectral range around the longitudinal plasmon. This adjustment could significantly enhance the ability to probe biomolecular interactions through LSPR tracking by minimizing laser interference.

In summary, we have introduced a depolarized forward light scattering (DFLS) methodology specifically designed for the analysis of thin biomolecular layers on anisotropic gold nanoparticles. The DFLS technique, implemented in a transmission configuration, allows for precise tracking of the nanoparticles’ rotational diffusion without interference from translational Brownian motion, providing subnanometer sensitivity to changes in biomolecular layer thickness. This high sensitivity is particularly advantageous for monitoring adsorption kinetics and conformational changes of biomolecules in various environments, which is critical for optimizing nanoparticle functionality in biological systems.

DFLS effectively captured the dynamics of functional coatings, such as CTAB, SAMs, and PEG, including temperature-dependent transitions such as the dissociation of the CTAB layer above 300 K. These small temperature-induced transitions in biomolecular coatings are challenging to detect using extinction spectroscopy, as the subtle LSPR shifts associated with these interactions are often obscured by significant bulk refractive index changes in the surrounding medium.

We further showed DFLS’s ability to monitor biomolecular adsorption kinetics, specifically for PEG and BSA, and validated the equilibrium affinity constants through complementary LSPR measurements. Additionally, DFLS revealed the influence of ionic strength on PEG layer stability, where higher NaCl concentrations caused the dissociation of noncovalently bound PEG molecules.

One of the primary advantages of DFLS is its focus on rotational diffusion without interference from translational motion. This allows for the precise detection of molecules adsorbed at the ends of nanorods, where changes in rotational drag are most pronounced. This end-specific sensitivity is beneficial when studying interactions that predominantly occur at these sites, such as ligand binding or functionalization processes that target the nanorod tips.

However, the technique has limitations that must be considered. First, the effectiveness of DFLS depends on the optical and scattering properties of the nanoparticles. Very small nanoparticles may not scatter enough light to produce measurable DFLS signals, limiting the utility of the method to particles above a certain size threshold and those made of plasmonic materials. Second, higher concentrations of nanorods can lead to multiple scattering events that can obscure the true rotational diffusion signal by introducing additional scattering paths that complicate data interpretation. To mitigate this, optimization of the nanoparticle concentration is necessary, balancing the need for a strong signal against the risk of multiple scattering. Lastly, although DFLS can yield absolute measurements of biomolecular layer thickness, factors such as variations in the nanoparticle size, shape, and aggregation state can introduce uncertainties in absolute thickness determinations. Consequently, DFLS is particularly well-suited for monitoring dynamic processes in which relative changes in layer thickness over time are of primary interest, such as adsorption kinetics or conformational shifts.

The DFLS method holds significant potential for *in vivo* applications due to its ability to provide real-time, label-free measurements of molecular interactions and dynamics with high sensitivity. However, translating DFLS to *in vivo* applications requires further optimization, including improvements in signal-to-noise ratios, enhancements in penetration depth for measurements in tissue environments, and integration of the method with optical setups tailored for biological imaging. With these technical challenges addressed in future developments, DFLS could become a powerful tool for probing molecular-scale interactions and characterizing the local environment within living organisms.

Overall, DFLS proved to be a versatile and powerful technique for real-time monitoring of the thickness and dynamics of biomolecular layers, providing valuable insights into the behavior of surface coatings under various environmental conditions. These findings underscore the potential of DFLS in applications such as nanoparticle-based drug delivery, biosensing, and the broader field of nanomedicine, where precise control and an understanding of surface interactions are critical.

## Experimental Methods

### DFLS Experimental Setup and Measurements

The beam of a single-mode diode laser (λ = 785 nm, Toptica) was expanded to a width of ∼3 mm, collimated, and linearly polarized by a Glan–Thompson polarizer (Figure 1a). The beam was focused using a lens with a focal length of *f* = 7 cm (L1) and directed into a 1 cm cuvette equipped with temperature control (Cary, Agilent) and a stirrer. An overlapping illumination path with collimated white light (halogen lamp HL-2000, Ocean Optics) was used for extinction spectroscopy. The transmitted light was collected by a second *f* = 7 cm lens (L2) and divided by a nonpolarizing dichroic beam splitter with a cutoff wavelength of 750 nm (Semrock). Transmitted white light with wavelength λ < 750 nm reflected from the dichroic beam splitter was collected by a fiber-coupled spectrometer (BWTek) for analysis of nanorod extinction spectra with 1 s accumulation time. Laser light transmitted through the dichroic beam splitter passes through a second Glan–Thompson polarizer oriented perpendicularly to the incident polarization and collected by a fiber-coupled photomultiplier (PMT, Becker and Hickel) connected to a digital autocorrelator for DFLS analysis based on the measured autocorrelation function (ACF). The sampling volume of the setup is approximately 0.1 mm^3^, the ACF integration time was 5 s, and the effective numerical apertures of the illumination and collection optics correspond to scattering angles <1.25°, i.e., *q*^2^ < 5.4 × 10^10^ m^–2^. Considering the typical values of *D_T_* ∼ 1–20 μm^2^ s^–1^ reported for nanorods of length L ∼ 40–565 nm,^28^ Γ_*R*_/Γ_*T*_ > 10^3^–10^5^. Therefore, the contributions of translational diffusion to ACF are negligible compared to the contribution of rotational Brownian motion.

### Data Analysis

For a polydisperse suspension of nanorods, the depolarized field correlation function, for *q* = 0 can be expressed as the Laplace transform of the probability density function *P*(Γ_*R*_) that describes the variation in relaxation rate (see SI for more details of derivation):^47^
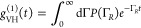
3*P*(Γ_*R*_) can thus in principle be determined through an inverse Laplace transform of the measured correlation function. Unfortunately, this problem is mathematically ill-posed, resulting in many possible solutions, unless additional constraints are imposed. Here we used the constrained regularization method for inverting data (CONTIN),^40,41^ as recently implemented in MATLAB code by Marino.^42^ To limit the number of possible solutions, a regularization strategy must be implemented. Here we have used the so-called L-curve criterion, which was recently shown to provide more reliable regularization than the method originally implemented in CONTIN.^45^

### Fabrication and Functionalization of Gold Nanorods

Nanorods (NRs) of nominal lengths between 65 and 190 nm were prepared by a seed-mediated growth method using cethydriltrimethylammonium bromide (CTAB) as the stabilizing agent.^35,36^ Before use, the suspension was centrifuged, the supernatant was removed, and the NRs were resuspended in aqueous solution of 1 mM CTAB. All samples were analyzed by scanning electron microscopy (SEM) (SI, Figure S2) and spectrophotometry (SI, Figure S3) to determine size variation (SI, Table T1) and average optical properties, respectively. The NR lengths and aspect ratios are such that the longitudinal plasmon resonance wavelength is close to the laser wavelength, λ = 785 nm, used in the DFLS measurements. The concentration of NR2 was measured with nanoparticle tracking analysis (NTA) that yielded the concentration of the stock solution at 10^12^ particles/mL. Citrate-stabilized spherical gold particles with a diameter of 100 nm were purchased from Sigma-Aldrich.

The NRs were functionalized with 11-mercaptoundecanoyl-hexaethylene glycoloic acid (11-MU-EG_6_-AA, Prochimia) as follows: Alkanethiols were dissolved in ethanol at a concentration of 10 mM and then mixed with the NR solution to a final concentration of 0.5 mM, corresponding to a CTAB:thiol ratio of approximately 1:4.^48^ The NR solution was incubated in a fridge overnight, centrifuged at high speed to remove the supernatant, resuspended in water, and finally centrifuged at low speed to remove any aggregated nanorods.

To create PEGylated nanorods, thiolated PEG with molecular weight of 2 or 5 kDa (PEG_2kDa_ and PEG_5kDa_, respectively) were added into the NR solutions to a final concentration of 1 mg/mL. The solutions were incubated overnight, centrifuged, and redispersed in 10× diluted PBS buffer (10 mM phosphate buffer, 140 mM NaCl, and 3 mM KCl, pH 7.4 at 25 °C; Sigma-Aldrich).

### T-matrix Calculations

The T-matrix method was used to calculate the optical response and, hence, the scattering cross sections of the gold nanoparticles. This makes it possible to handle scattering off nonspherical particles using the same kind of multipolar basis functions as in Mie theory for particles possessing spherical symmetry. The difference is that the response of a nonspherical particle involves interaction between different multipoles. The calculations, particularly the steps taken to average scattering cross sections over different particle orientations, are described in more detail in the Supporting Information.

## Supporting Information Available

Description of T-matrix calculations, DFLS theory, geometrical and scattering properties of gold nanorods, and supplementary figures to detection of biomolecular layers (PDF)
Transparent Peer Review report available (PDF)
