# Supplementary material for: Depolarized Forward Light Scattering for Subnanometer Precision in Biomolecular Layer Analysis on Gold Nanorods
Source: J Phys Chem Lett. 2025 Jan 27;16(5):1288–95. doi: 10.1021/acs.jpclett.4c02956 (PMC11808774; doi:10.1021/acs.jpclett.4c02956)
Supplement: Supplementary file 1 — jz4c02956_si_001.pdf [file jz4c02956_si_001.pdf]

SUPPORTING INFORMATION

Depolarized Forward Light Scattering for Sub-Nanometer Precision in Biomolecular Layer Analysis on Gold Nanorods

*Peter Johansson[1], Mikael Käll[2], Hana Šípová-Jungová[2],**

*Corresponding author: hana.jungova@chalmers.se

AUTHOR ADDRESS

[1]School of Science and Technology, Örebro University, 701 82 Örebro, Sweden

[2]Department of Physics, Chalmers University of Technology, 412 96 Göteborg, Sweden.

Outline:

T-matrix calculations of the scattering cross-sections 1

Averaging the scattering cross-section over particle orientations 1

Dynamic light scattering theory 3

Effect of particle heating on the rotational diffusion 4

Geometrical and scattering properties of gold nanorods 7

Detection of biomolecular layers 10

T-MATRIX CALCULATION OF THE SCATTERING CROSS SECTION

We use the T-matrix method[1, 2] to calculate the scattering cross section of the nanoparticles (nanorods). The use of this method goes back to Waterman, but has since been refined and used in many different contexts. The T-matrix method is an extension of Mie theory allowing one to treat the response of a non-spherical nanoparticle in terms of incident and scattered electric and magnetic multipoles. In this extended framework an incident field in one multipole channel yields scattering into several other channels.

We specifically deal with particles that have cylindrical symmetry as well as inversion symmetry such as spheroids and circular cylinders capped by hemispheres. In a coordinate system fixed on the scatterer with the z axis aligned with the cylindrical symmetry axis of the particle, the electric field of the incident radiation in the surrounding medium can be written

$$\vec{E}_{\mathrm{inc}}(\vec{r}) = \sum_{\tau lm} a_{\tau lm} \vec{\psi}^{(R)}_{\tau lm}(\vec{r}) \tag{1}$$

and the field of the scattered wave is

$$\vec{E}_{\mathrm{sc}}(\vec{r}) = \sum_{\tau lm} b_{\tau lm} \vec{\psi}^{(O)}_{\tau lm}(\vec{r}). \tag{2}$$

In these expressions τ denotes the type of multipole ($\tau = 1$ stands for a magnetic multipole, $\tau = 2$ for an electric multipole), while l and m are the usual quantum numbers associated with spherical symmetry. The superscripts (R) and (O) indicate whether the basis function $\vec{\psi}$ describes an incident, regular, wave or an outgoing wave.

The basis functions in Eqs. (1) and (2) are given by

$$\vec{\psi}^{(R,O)}_{1lm} = \vec{X}_{lm}(\theta, \varphi) z_l(kr) \tag{3}$$

and

$$\vec{\psi}^{(R,O)}_{2lm} = k^{-1} \nabla \times \left\{ \vec{X}_{lm}(\theta, \varphi) z_l(kr) \right\}, \tag{4}$$

where k is the wave number in the medium, $k = \sqrt{\varepsilon_{\mathrm{rel}}}\omega/c$, with $\varepsilon_{\mathrm{rel}}$ the dielectric function of the medium, ω the angular frequency of the light, and c the speed of light in vacuum. $z_l(kr)$ either denotes a spherical Bessel function (regular, incident, wave) or a spherical Hankel function (outgoing wave) and the vector spherical harmonic $\vec{X}_{lm}(\theta, \varphi)$ can be expressed using the ordinary spherical harmonic $Y_{lm}(\theta, \varphi)$ and the angular momentum operator $\hat{L} = -i\vec{r} \times \nabla$ as

$$\vec{X}_{lm} = \hat{L} Y_{lm} / \sqrt{l(l+1)}. \tag{5}$$

The T matrix provides us with a relation between the coefficients for the incident multipoles and the scattered ones,

$$\vec{b} = \overleftrightarrow{T} \vec{a} \quad \Longleftrightarrow \quad b_{\tau lm} = \sum_{\tau' l' m'} T_{\tau lm, \tau' l' m'} a_{\tau' l' m'}. \tag{6}$$

The T matrix depends on the size and shape of the particle, the dielectric properties of the particle and the surrounding medium, and the frequency of the electromagnetic field. In the calculations here we have used the dielectric function of gold from Johnson and Christy[3]. In the case of a spherical particle, the T matrix is diagonal; then there is no cross-coupling between different multipoles. The particles we consider here do not have spherical symmetry but they still have cylindrical as well as inversion symmetry, Hence, in a coordinate system in which the z axis is aligned with the symmetry axis $\overleftrightarrow{T}$ is diagonal in the azimuthal quantum number m. Moreover, only multipoles with equal parity interact.

In a practical calculation the sums over angular momenta l have to be truncated at some point. For the calculations presented here we reach full convergence already when using a maximum l (l_{max}) of 6.

AVERAGING THE SCATTERING CROSS SECTION OVER PARTICLE ORIENTATIONS

We now use T-matrix theory to calculate the differential scattering cross section averaged over all possible particle orientations.

To this end we expand the incident and scattered radiation fields the same way as in Eqs. (1) and (2), however, now in a laboratory coordinate system (still with the origin at the particle center). The T matrix $\overleftrightarrow{T}^L$ in this coordinate system is determined by a rotational transformation

$$\overleftrightarrow{T}^L = \overleftrightarrow{D}(\alpha,\beta,0)\overleftrightarrow{T}^B\overleftrightarrow{D}^\dagger(\alpha,\beta,0), \implies \vec{b}^L = \overleftrightarrow{D}(\alpha,\beta,0)\overleftrightarrow{T}^B\overleftrightarrow{D}^\dagger(\alpha,\beta,0)\vec{a}^L. \tag{7}$$

where $\vec{b}^L$ and $\vec{a}^L$ are multipole expansion coefficients in the laboratory system, $\overleftrightarrow{T}^B$ is the T matrix in the body-fixed coordinate system, $\overleftrightarrow{D}$ is a Wigner rotation matrix. The particle orientation, the direction the particle symmetry axis points in, determines the Euler angles β and α.

Looking at a particular element of $\overleftrightarrow{T}^L$, we have

$$T^L_{\tau l m \tau' l' m'} = \sum_\mu D^l_{m\mu}(\alpha,\beta,0) T^\mu_{\tau l \tau' l'} [D^\dagger(\alpha,\beta,0)]^{l'}_{\mu m'} \tag{8}$$

where now $T^\mu_{\tau l \tau' l'}$ refers to the body T matrix, which is diagonal in the azimuthal quantum number, μ. This expression can be simplified using the Clebsch-Gordan series[4] for the product of Wigner matrix elements yielding

$$T_{\tau l m \tau' l' m'} = \sum_J (-1)^m (ll'; m, -m'|J, m-m') \mathcal{T}^{(J)}_{\tau l \tau' l'} \sqrt{\frac{4\pi}{2J+1}} Y_{Jm'-m}(\beta,\alpha). \tag{9}$$

Here $(ll'; m, -m'|J, m-m')$ is a Clebsch-Gordan coefficient, while the reduced scattering matrix $\mathcal{T}$ describing the scattering properties of the particle without any reference to its orientation is given by

$$\mathcal{T}^{(J)}_{\tau l \tau' l'} = \sum_\mu (-1)^\mu T^\mu_{\tau l \tau' l'} (ll'; \mu, -\mu|J, 0). \tag{10}$$

For an object of spherical symmetry $\mathcal{T}^{(J)}$ vanishes for $J \neq 0$. In this case there will be no cross-polarized scattering. For the particles that we study here the cross-polarized scattering originates from the $\mathcal{T}^{(2)}$ coefficients (related to the dipolar response of the particles) while the anisotropy associated with higher J values (4, 6, . . .) only give completely negligible contributions to the scattering.

The ratio between the scattered field, propagating in a specific direction and with a specific polarization, and the incident field E_0 depends on the particle orientation and as such it can be expanded in terms of spherical harmonics in orientational space (β, α),

$$(E_{\rm sc}/E_0) = \frac{e^{ikr}}{kr} \sum_{JM} s_{JM}(\hat{r}, \hat{\varepsilon}_{\rm out}, \hat{k}, \hat{\varepsilon}_{\rm in}) Y_{JM}(\beta,\alpha). \tag{11}$$

where the expansion coefficients s_{JM} are given by

$$s_{JM} = \sqrt{\frac{4\pi}{2J+1}} \sum_{\tau l m \tau' l'} (-1)^m \, (ll'; m, -M-m|J, -M) \, u_{\tau l m} \, \mathcal{T}^{(J)}_{\tau l \tau' l'} \, a_{\tau' l' M+m}, \tag{12}$$

and depend on the propagation direction $\hat{k}$ and polarization direction $\hat{\varepsilon}_{\rm in}$ of the incident light as well as the propagation direction $\hat{r}$ and polarization direction $\hat{\varepsilon}_{\rm out}$ of the scattered light. In Eq. (12) $a_{\tau l m}$ and $u_{\tau l m}$ are coupling coefficients between a multipole and an incident plane wave and a scattered plane wave, respectively. The coupling coefficients to the incident wave can be found from

$$a_{\tau l m} = \sum_{s=-1}^{1} 4\pi i^l (l1; m-s, s|l, m) \left[\vec{\xi}^*_s \cdot \hat{\eta}_\tau\right] (-1)^{m-s} Y_{l,s-m}(\hat{k}), \tag{13}$$

where

$$\hat{\eta}_1 = \hat{\varepsilon}_{\rm in}, \text{ and } \hat{\eta}_2 = i\hat{k} \times \hat{\varepsilon}_{\rm in}, \tag{14}$$

with $\hat{k}$ denoting the propagation direction of the incident light, $\hat{\varepsilon}_{\rm in}$ its polarization, while

$$\hat{\xi}_0 = \hat{z}, \qquad \hat{\xi}_{\pm 1} = \mp(\hat{x} \pm i\hat{y})/\sqrt{2}. \tag{15}$$

are circular basis unit vectors. The coupling to the scattered wave, propagating in the direction of the unit vector $\hat{r}$, and polarized along $\hat{\varepsilon}_{\text{out}}$ is found from

$$u_{1lm} = (-i)^{l+1} \vec{X}_{lm}(\hat{r}) \cdot \hat{\varepsilon}_{\text{out}}, \quad \text{and} \quad u_{2lm} = (-i)^{l} \left[\hat{r} \times \vec{X}_{lm}(\hat{r}) \right] \cdot \hat{\varepsilon}_{\text{out}}. \tag{16}$$

The differential scattering cross section is found by evaluating the Poynting vector in the scattered far field

$$S = \frac{1}{2} \sqrt{\varepsilon_{\text{rel}}} \varepsilon_0 c |E_{sc}|^2, \tag{17}$$

which then yields the scattered power per unit solid angle, $dP/d\Omega = Sr^2$. The differential scattering cross section $d\sigma/d\Omega$ is then the ratio between the differential power and the incident power,

$$S_{\text{in}} = \frac{1}{2} \sqrt{\varepsilon_{\text{rel}}} \varepsilon_0 c |E_0|^2, \tag{18}$$

hence for a specific particle orientation we get

$$\frac{d\sigma}{d\Omega}(\beta, \alpha) = r^2 \frac{S}{S_{in}} = \frac{1}{k^2} \left| \sum_{JM} s_{JM} Y_{JM}(\beta, \alpha) \right|^2 \tag{19}$$

Averaging this over all orientations then yields

$$\left\langle \frac{d\sigma}{d\Omega} \right\rangle = \frac{1}{4\pi} \int_0^{\pi} \sin\beta d\beta \int_0^{2\pi} d\alpha \, \frac{d\sigma}{d\Omega}(\beta, \alpha) = \frac{1}{4\pi k^2} \sum_{JM} |s_{JM}|^2. \tag{20}$$

DYNAMIC LIGHT SCATTERING THEORY

In dynamic light scattering (DLS) experiments, the scattering intensity I is measured over time and converted into a normalized autocorrelation function (ACF) $g^{(2)}(t)$,

$$g^{(2)}(t) = \frac{\langle I(t+t_0) I(t_0) \rangle}{\langle I(t_0) I(t_0) \rangle} \tag{21}$$

where t is the time delay and the averaging refers to the initial time t_0. This correlation function can be converted into the corresponding autocorrelation function for the scattered electric field $g^{(1)}(t)$ using the Siegert relation

$$g^{(2)}(t) = 1 + b|g^{(1)}(t)|^2. \tag{22}$$

For noninteracting monodisperse samples of sufficiently small particles, the depolarized field autocorrelation function $g^{(1)}_{VH}(t)$ can be expressed as[5]

$$g^{(1)}_{VH}(t) = e^{-(\Gamma_T + \Gamma_R)t}, \tag{23}$$

with exponents determined by the translational and rotational diffusion coefficients, D_T and D_R, as

$$\Gamma_T = q^2 D_T \quad \text{and} \quad \Gamma_R = 6 D_R. \tag{24}$$

Here q is the absolute value of the scattering wave vector, set by the scattering angle θ,

$$q = \frac{4\pi n}{\lambda} \sin(\theta/2), \tag{25}$$

λ is the light wavelength in vacuum, and n the refractive index of the liquid. Equations (23) and (24) show that the rotational diffusion contributes to the decay of the autocorrelation of the depolarized scattering irrespective of the scattering angle, whereas the effects of translational diffusion vanish for small scattering angles, $\theta \approx 0$. Hence by using a forward scattering geometry one can isolate the effects of the rotational Brownian motion to the depolarized dynamic light scattering (DDLS) signal. The measured intensity autocorrelation function of depolarized scattering can then be expressed as

$$g^{(2)}_{VH}(t) = 1 + b e^{-t/\tau_0} = 1 + b e^{-12 D_R t}, \tag{26}$$

where τ_0 is the autocorrelation function decay time.

For a particle shaped like a prolate spheroid the rotational diffusion constant can be written as

$$D_R = \frac{k_B T}{3\pi\eta g L^3}, \tag{27}$$

where k_B is the Boltzmann constant, T the temperature, L the length of the nanorod, η the temperature-dependent dynamic viscosity of the fluid[6], and g is a geometrical factor that depends on the eccentricity of the nanorod[7, 8].

EFFECTS OF PARTICLE HEATING ON THE ROTATIONAL DIFFUSION

The illumination of the nanoparticles lead to heating. This in turn also changes the properties of the surrounding fluid, including its viscosity. As discussed by Ruijgrok *et al.*[9], the rate of rotational diffusion is largely governed by an effective temperature approximately equal to the particle temperature. Using a power balance argument, it is easy to see that the thermal equilibration between a spherical nanoparticle of radius R and the surrounding fluid takes place on a time scale

$$\tau_{\rm eq} = \frac{c_{\rm Au}\rho R^2}{3\kappa}, \tag{28}$$

where $c_{\rm Au}$ and ρ are the specific heat capacity and density, respectively, of the gold nanoparticle and R is its radius, whereas κ denotes the heat conductivity of the fluid. For a gold particle of radius 75 nm in water we get $\tau_{\rm eq} \approx 7.8\,\rm ns$, hence considerable shorter than the time scales relevant to the rotational diffusion. In view of this we have a rotational diffusion problem where the diffusion constant depends on the particle orientation. This means that there may be modifications of the relation between the rotational diffusion constant and the autocorrelation function decay time. Here we investigate these aspects.

We can write the previously introduced normalized intensity-intensity correlation function $g^{(2)}(t)$ as

$$g^{(2)}(t) = \frac{\langle E^*_{sc,N}(t)E_{sc,N}(t)E^*_{sc,N}(0)E_{sc,N}(0)\rangle}{|\langle E^*_{sc,N}(0)E_{sc,N}(0)\rangle|^2}, \tag{29}$$

where t still denotes the time delay, while the averaging now is done over all possible initial configurations at one time, $t = 0$. We would like to underscore that the scattered fields here result from scattering from N particles. Provided that the number of particles is large $N >> 1$ and the coherence between the fields scattered off different particles is limited we get

$$\langle E^*_{sc,N}(t)E_{sc,N}(t)E^*_{sc,N}(0)E_{sc,N}(0)\rangle = N^2\left[|\langle E^*_{sc}(0)E_{sc}(0)\rangle|^2 + |\langle E^*_{sc}(t)E_{sc}(0)\rangle|^2\right] \tag{30}$$

while

$$\langle E^*_{sc,N}(0)E_{sc,N}(0)\rangle = N|\langle E^*_{sc}(0)E_{sc}(0)\rangle|^2 \tag{31}$$

where the scattering amplitudes E_{sc} now refer to the fields generated by one particle. This means that

$$g^{(2)}(t) = 1 + \frac{|\langle E^*_{sc}(t)E_{sc}(0)\rangle|^2}{|\langle E^*_{sc}(0)E_{sc}(0)\rangle|^2}. \tag{32}$$

The ratio in the second term can be related to the quantities used in evaluating the average scattering cross section. Hence, in view of Eqs. (11) and (20) we can write

$$g^{(2)}(t) = 1 + \left(\frac{\langle\sum_{J'M'JM} s^*_{J'M'}Y^*_{J'M'}(\Omega(t))Y_{JM}(\Omega(0))s_{JM}\rangle}{(4\pi)^{-1}\sum_{JM}|s_{JM}|^2}\right)^2, \tag{33}$$

where Ω is a solid angle variable representing the particle orientation, the angles (β,α) used above.

To calculate the expectation value in the numerator we need to know the orientation of a particle at two different times. We express $< Y^*_{J'M'}(\Omega(t))Y_{JM}(\Omega(0)) >$ as

$$< Y^*_{J'M'}(\Omega(t))Y_{JM}(\Omega(0)) >= \int d\Omega \int \frac{d\Omega_0}{4\pi} Y^*_{J'M'}(\Omega)\,G(\Omega,\Omega_0,t)\,Y_{JM}(\Omega_0), \tag{34}$$

where $G(\Omega, \Omega_0, t)$ is a Green's function describing the probability for a particle oriented along Ω_0 at time $t = 0$ to have orientation Ω at time t.

The Green's function solves the diffusion equation on the unit sphere,

$$\frac{\partial G}{\partial t}(\Omega, \Omega_0, t) = \nabla \cdot \left[D(\Omega)\nabla G(\Omega, \Omega_0, t)\right]. \tag{35}$$

Here ∇ operates on the (two-dimensional) unit sphere, hence $\nabla^2 = -\hat{L}^2$, and we will assume that the orientational dependence of the diffusion coefficient is given by

$$D(\Omega) = D_0 + D_2 Y_{20}(\Omega), \tag{36}$$

that is the diffusion coefficient has a constant part and a contribution that depends on the particle orientation as $\cos^2\beta$ due to heating of the particle when it is aligned with the polarization of the incident light.

We represent the Green's function in terms of spherical harmonics as,

$$G(\Omega, \Omega_0, t) = \sum_{lml'm'} Y_{l'm'}(\Omega) G_{l'm'lm}(t) Y^*_{l'm'}(\Omega_0). \tag{37}$$

This expression can be inserted into Eqs. (34) and (33) in a straightforward way which yields

$$g^{(2)}(t) = 1 + \left(\frac{\sum_{J'M'JM} s^*_{J'M'} G_{J'M'JM}(t) s_{JM}}{\sum_{JM} |s_{JM}|^2} \right)^2. \tag{38}$$

It still remains to determine the time dependence of $G_{l'm'lm}(t)$. At $t = 0$ we have the initial condition

$$G_{l'm'lm}(0) = \delta_{l',l}\delta_{m'm}. \tag{39}$$

Furthermore, the cylindrical symmetry of the diffusion coefficient means that G remains diagonal in m also for $t > 0$, $G_{l'm'lm}(t) = \delta_{m',m} G^{(m)}_{l'l}(t)$. By inserting the angular representation of G, Eq. (37), into the diffusion equation we get

$$\frac{dG^{(m)}_{l'l}}{dt} = \sum_{l''} M^{(m)}_{l'l''} G^{(m)}_{l''l}, \tag{40}$$

where the matrix M, with elements

$$M^{(m)}_{l'l''} = \langle l'm|\nabla D(\Omega)\nabla|l''m\rangle = \int D(\Omega)\, \left[-\nabla Y_{l'm}(\Omega)\right]^* \cdot \left[\nabla Y_{l''m}(\Omega)\right]\, d\Omega, \tag{41}$$

can be diagonalized yielding real eigenvalues that set the rate of decay of the different angular components due to rotational diffusion.

As in the case of a constant D, there is a zero eigenvalue (which means that $G^{(0)}_{00} \equiv 1$ at all times) also when $D(\Omega)$ depends on the particle orientation. But the second lowest eigenvalue, which is $J(J+1)D = 6D$ with $J = 2$ in the case of a diffusion constant independent of particle orientation, splits and shifts with an orientation-dependent diffusion constant. However, as we show with a concrete example below these changes do not lead to very different autocoreelation functions, even when the temperature differences are considerably higher than in our experiments.

Figure S1 shows the behavior of $g^{(2)}(t)$ in a few different situations. We assume that the diffusion coefficient depends on the orientation as in Eq. (36) and determine the diffusion coefficient based on the water temperature in four different cases: (i) $T = T_l = 293\,\mathrm{K}$ for all orientations, (ii) $T = T_h = 323\,\mathrm{K}$ for all orientations (iii) orientation-dependent temperature, $T = T_l = 293\,\mathrm{K}$ for horizontally (orthogonal to the incident light polarization) oriented particles and $T = T_h = 323\,\mathrm{K}$ for vertically oriented particles, and, finally, (iv) $T = 303\,\mathrm{K}$ for all particle orientations. We see that the results for $g^{(2)}(t)$ varies monotonically with the temperature, and in the case of an orientation-dependent diffusion constant the result agrees rather well with what one would get with a constant, spherical average, temperature $T = (T_h + 2T_l)/3$.

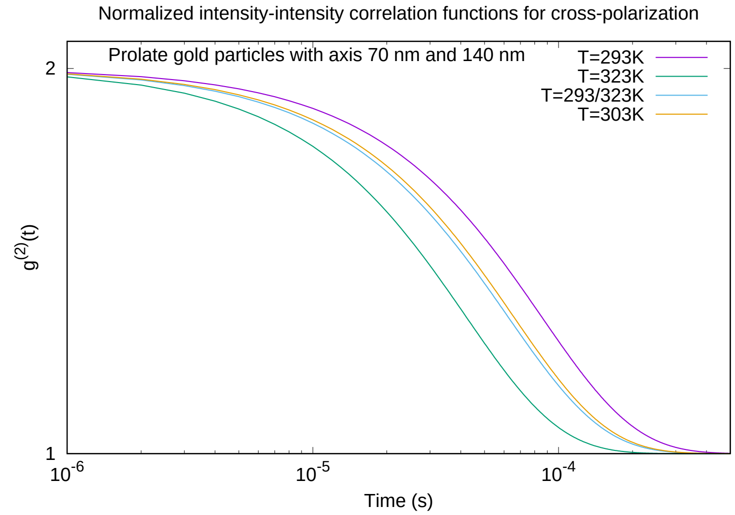

Fig. S1. Calculated time dependence for the function $g^{(2)}(t)$, the normalized intensity-intensity correlation function in Eq. (33) for cross-polarized scattering and with diffusion constants corresponding to three different constant temperatures and an orientation-dependent temperature and diffusion constant (the T293/323 case).

---

[1] Waterman, P. C. Symmetry, unitarity, and geometry in electromagnetic scattering. *Phys. Rev. D* **1971**, 3 (4) 825–839. DOI: 10.1103/PhysRevD.3.825.

[2] Peterson, B.; Ström, S. T matrix for electromagnetic scattering from an arbitrary number of scatterers and representations of E(3). *Phys. Rev. D* **1973**, 8 (10) 3661–3678. DOI: 10.1103/PhysRevD.8.3661.

[3] Johnson, P. B.; Christy, R. W.; Optical constants of the noble metals. *Phys. Rev. B* **1972**, 6 (12) 4370–4379. DOI: 10.1103/PhysRevB.6.4370.

[4] Sakurai, J. J.; Napolitano, J.; *Modern Quantum Mechanics*, 3rd ed.; Cambridge University Press, **2020**, Ch. 3.

[5] Balog, S.; Rodriguez-Lorenzo, L.; Monnier, C. A.; Michen, B.; Obiols-Rabaza, M.; Casal-Dujat, L.; Rothen-Rutishauser, B.; Petri-Fink, A.; Schurtenberger, P.; Dynamic depolarized light scattering of small round plasmonic nanoparticles: when imperfection is only perfect. *J. Phys. Chem. C* **2014** 118 (31) 17968–17974. DOI: 10.1021/jp504264f.

[6] Fogel'son, R.; Likhachev, E.; Temperature dependence of viscosity. *Technical Physics* **2001**, 46 (8) 1056–1059.

[7] Šipová-Jungová, H.; Shao, L.; Odebo-Länk, N.; Andrén, D.; Käll. M. Photothermal DNA release from laser-tweezed individual gold nanomotors driven by photon angular momentum. *ACS Photonics* **2018**, 5 (6) 2168–2175. DOI: 10.1021/acsphotonics.8b00034.

[8] Kong, D.; Lin, W.; Pan, Y.; Zhang, K. Swimming motion of rod-shaped magnetotactic bacteria: the effects of shape and growing magnetic moment. *Frontiers in Microbiology* **2014**, 5. DOI: 10.3389/fmicb.2014.00008.

[9] Ruijgrok, P. V.; Verhart, N. L.; Zijlstra, P.; Tchebotareva, A. L.; Orrit, M. Brownian fluctuations and heating of an optically aligned gold nanorod. *Phys. Rev. Lett.* **2011** 107, 037401. DOI: 10.1103/PhysRevLett.107.037401.

GEOMETRICAL AND SCATTERING PROPERTIES OF GOLD NANORODS

Gold nanorods used in this study were analyzed by standard spectrophotometry to determine their optical properties and by scanning electron microscopy (SEM) to assess their size variation. The average length and width of the nanorods are provided in Table T1.

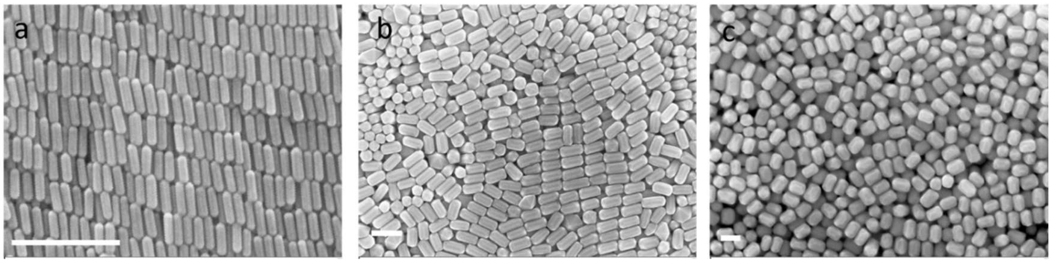

Fig S2: *SEM images of nanorods synthetized with seeded mediated growth. Scale bar represents 200nm. a) NR1, b) NR2, c) NR3.*

	L [nm]	d [nm]
NR1	65 (6)	21 (2)
NR2	140(9)	70 (4)
NR3	190 (18)	127 (11)

Tab. T1: *Parameters of gold nanorods used in this study, including length (L) and diameter (d). The lengths and diameters were measured from SEM images.*

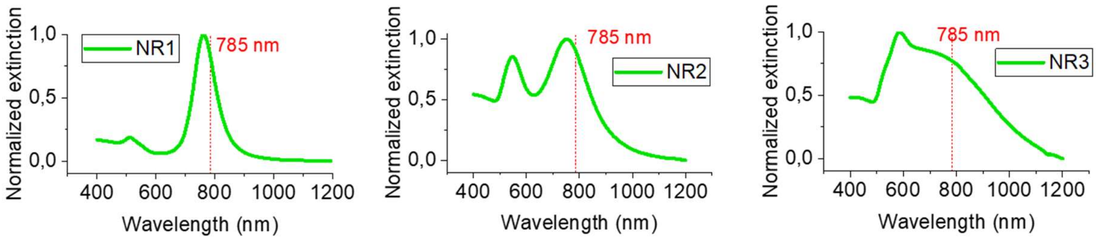

Fig. S3: *UV–Vis extinction spectra of nanorods summarized in Table S1. The spectra show a low-energy peak corresponding to the nanorod longitudinal localized surface plasmon resonance (LSPR) and a high-energy peak corresponding to the transverse LSPR. The dashed line indicates the laser wavelength (785 nm) used in the experiments. Spectra were obtained using a Varian 3000 UV–Vis spectrometer with a 1 cm optical path length cuvette.*

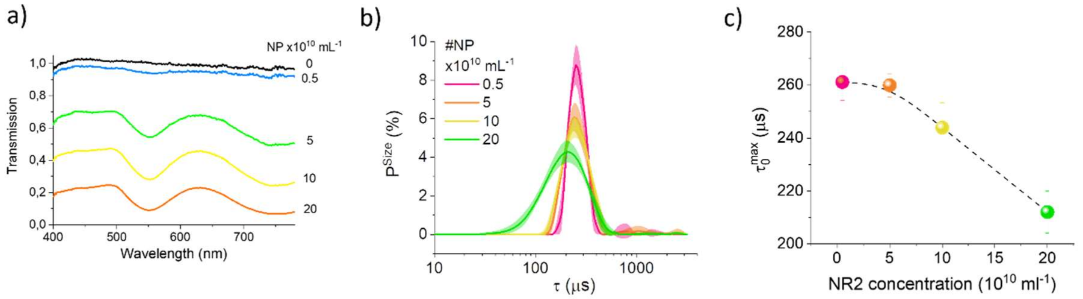

Fig. S4. *The effect of nanorod concentration on the DFLS signal and extinction spectra a) Transmission spectra of diluted NR2 samples recorded simultaneously with the DFLS measurements b) decay time distributions* $P^{S}(\tau_0)$ *and c) distribution maxima at various NR concentrations.*

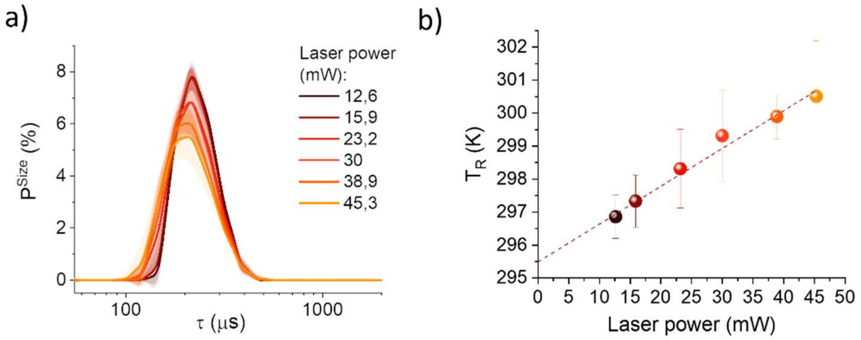

Fig. S5. *a) The effect of plasmonic heating and bulk temperature on calculated size distribution of NR2 suspension b) Decay time distribution variation with laser power and (b) temperature* T_R *calculated from* τ_{max} *of distributions in panel (a) with Eq. 1 in the main text. Dashed line represents linear fit (intercept at 295±0,1K and slope at 0,114±0,005 K/mW).*

L (nm)	λ_{VV}^{max}(nm)	λ_{VH}^{max} (nm)	λ_{VV+VH}^{max} (nm)	$\lambda_{\Delta_{VH}^{max}}^{max}$ (nm)	Δ_{VH}^{max} (%)	Δ_{VH}^{785} (%)
130	694,4±0,1	677,1±0,2	690,75± 0,05	624,1±0,4	27,5	14
140	726,5±0,1	707,7±0,3	722,57± 0,04	636,4±0,4	29,8	17
150	760,0±0,1	740,7±0,4	755,79±0,03	645,8±0,3	31,4	20

Tab. T2: *Light scattering parameters of nanorods, calculated using the T-matrix method, for widths of 70 nm and lengths of 130, 140, and 150 nm. Parameters include the spectral positions of maxima for co-polarized* (λ_{VV}^{max})*, depolarized* (λ_{VH}^{max})*, total scattering* (λ_{VV+VH}^{max}) *and depolarization ratio* ($\lambda_{\Delta_{VH}^{max}}^{max}$)*. The table also presents the depolarization ratio values at the maximum* Δ_{VH}^{max} *and at 785 nm wavelength* Δ_{VH}^{785}*.*

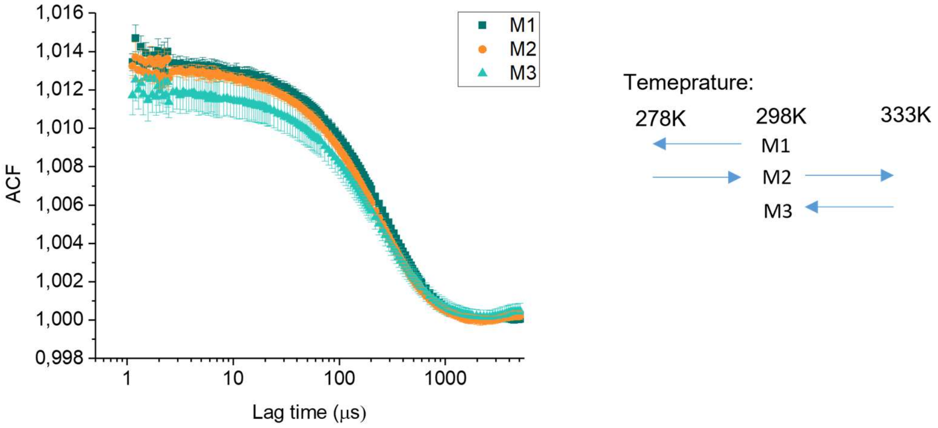

Fig. S6: *Depolarized autocorrelation functions (ACFs) of CTAB-coated NR2 measured at 298.15 K at three stages during the temperature scan shown in Figure 3 of the main text: at the beginning (M1), in the middle (M2), and at the end (M3). The schematic on the right illustrates the sequence in which measurements M1–M3 were taken. Each ACF is calculated as an average from 10 scans, with each scan lasting 5 seconds. Measurement M3 was taken after decreasing the temperature from 333 K and showed higher variability in scattering intensity between measurements, likely due to small bubbles in the solution formed at higher temperatures and vapor condensation. However, the average decay times of the ACFs for M2 and M3 are almost identical—260 ± 9 μs and 257 ± 8 μs, respectively, demonstrating that CTAB dissociation at higher temperature is reversible.*

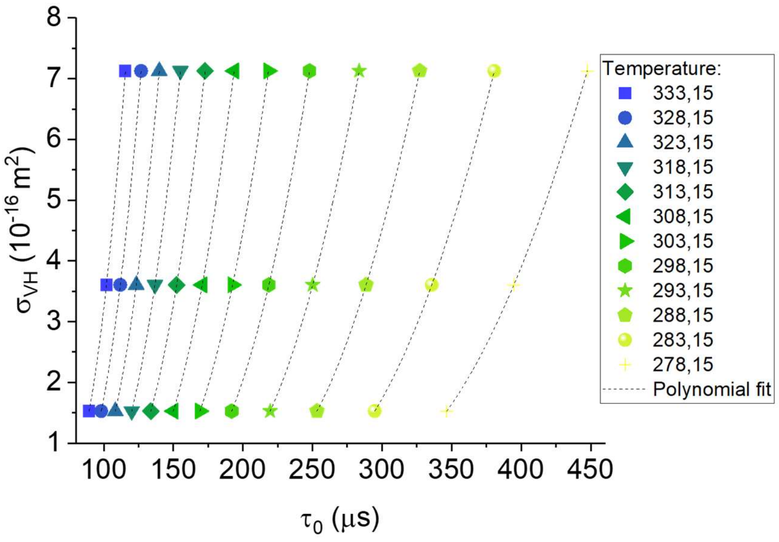

Fig. S7: σ_{VH} *at laser wavelength 785 nm as a function of* τ_0 *for NR2. The* τ_0 *was calculated using eq. (1) in the main text with values of L = 140 nm, d = 70 nm and water viscosity based on equation* $\eta(T) = \eta_0 \exp\{E/k_B(T + T')\}$, *where* η_0*=2.4152×10-5 Pa·s, E = 4.7428 kJ/mol and* T' *= -139.86 K are constants. Line indicates a fit with a 2nd order polynomial.*

DETECTION OF BIOMOLECULAR LAYERS

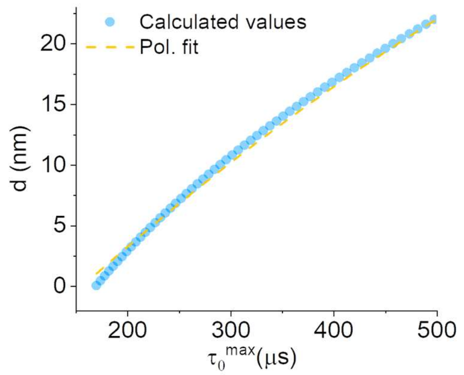

Fig. S8: *Calibration curve for biolayer thickness (d) using τ_0^{max} values and Eq. 1 in the main text at a constant temperature of 292 K. Third-order polynomial approximation is shown as a dashed line.*

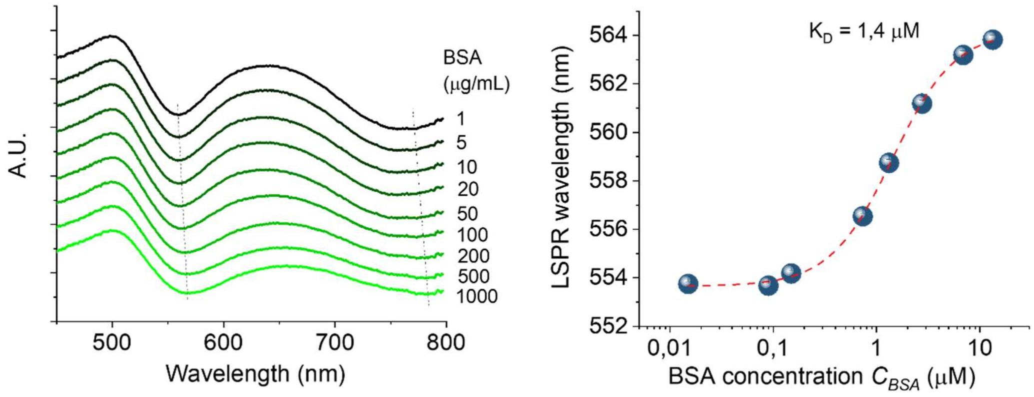

Fig. S9. *Examination of NR07 nanorods functionalized with PEG_{5kDa} across BSA concentrations using extinction spectroscopy. Left: Transmission spectra simultaneously recorded during the DDLS measurements shown in Fig. 4d. Right: Spectral positioning of the short LSPR. A dashed line delineates fit with Logistic function, producing an affinity constant of 1.4 µM – a result in agreement with the DDLS measurements.*
