# Supplementary material for: Depolarized Forward Light Scattering for Subnanometer Precision in Biomolecular Layer Analysis on Gold Nanorods
Source: J Phys Chem Lett. 2025 Jan 27;16(5):1288–95. doi: 10.1021/acs.jpclett.4c02956 (PMC11808774; doi:10.1021/acs.jpclett.4c02956)
Supplement: Supplementary file 2 — jz4c02956_si_002.pdf [file jz4c02956_si_002.pdf]

jz-2024-029566

Name: Peer Review Information for "Depolarized Forward Light Scattering for Sub-Nanometer Precision in Biomolecular Layer Analysis on Gold Nanorods"

First Round of Reviewer Comments

Reviewer: 1

Comments to the Author

Major Advance Reported:

The paper introduces Depolarized Forward Light Scattering (DFLS), which modifies traditional depolarized dynamic light scattering by using a transmission configuration. It isolates rotational diffusion signals from translational Brownian motion, combined with measurements near the longitudinal surface plasmon resonance of gold nanorods. This allows for real-time, in situ measurements of biomolecular layer thickness on nanoparticles with sub-nanometer precision.

Immediate Significance:

This work represents a methodological advance in nanoparticle characterization, providing a label-free tool for studying biomolecular interactions at nanoparticle surfaces. The technique enables real-time monitoring of surface modification processes and allows investigation of environmental effects such as temperature and ionic strength on surface coatings. As a complementary approach to existing characterization methods, it shows promise for applications in nanoparticle-based drug delivery systems.

Technical Suggestions:

1. The manuscript would benefit from a more detailed discussion of the technique's limitations, including minimum detectable layer thickness changes.
2. The reversibility of temperature-dependent changes in CTAB layer (Figure 3) should be addressed.
3. The organization of Figure 4d feels disconnected from previous panels and needs better integration.
4. The fundamental principles behind depolarized forward scattering advantages (how separating rotational diffusion signals from translational Brownian motion) need clearer explanation for broader accessibility.
5. The authors should provide quantitative comparisons with existing methods to better highlight the specific improvements achieved.
6. The biological interaction studies (Figure 4) could be expanded to better demonstrate the method's practical advantages.
7. The manuscript structure should be reorganized to better emphasize practical applications rather than technical validation.
8. Claims about real time kinetic measurements should be supported with actual time-resolved studies.

After addressing the above comments, the manuscript can be recommended for publication in The Journal of Physical Chemistry Letters.

Reviewer: 2

Comments to the Author

Depolarized Forward Light Scattering for SubNanometer Precision in Biomolecular Layer Analysis on Gold Nanorods

Peter Johansson, Mikael Käll, Hana Šípová-Jungová

Johansson et al. present a novel method, so-called "depolarized forward light scattering (DFLS)" for determining fast rotational motion of a nanorod, most importantly its interaction with biomolecules with a subnanometer precision by demonstrating detection of temperature-induced dissociation of CTAB layer on gold nanorods. In addition, they studied adsorption kinetics of PEG and BSA at different ionic strengths. The work is very interesting. The proposed method is promising for applications of nanoparticles-biomolecules interactions. The paper can be published as it is now. However, the authors may consider some of my following minor comments before its publication.

(1) The viscosity (η) in equation 1 is only considered to be as a function of temperature η (T) as temperature change is due to the laser heating. However, I think that it is more complex than that. Viscosity will also be as a function of time as the particles rotates and so its absorption of linear polarization will change and so the temperature-rise. This means that η is as η (T, t). In addition, the laser induced heating is a gradient profile and so, η is η (T, t, r) where r is the distance from the particle surface. Can the authors comment on this?

(2) For extinction spectroscopy, the authors used dichroic to separate the laser detection from white light. However, this blocks important information regarding the longitudinal plasmon. For nanoparticles aggregation or biomolecular interactions, extinction spectroscopy around longitudinal plasmon will be important. The authors can block only the narrow laser band with some filters and can detect the rest of the signal around the longitudinal plasmon. Can the authors comment on this?

(3) Can the temperature-induced CTAB dissociation also be observed by extinction spectroscopy, specifically by longitudinal plasmon-shift?

(4) Can the authors give some perspective in terms of in-vivo applications of DFLS method?

Author's Response to Peer Review Comments:

Response to reviewers:

Reviewer 1

The paper introduces Depolarized Forward Light Scattering (DFLS), which modifies traditional depolarized dynamic light scattering by using a transmission configuration. It isolates rotational diffusion signals from translational Brownian motion, combined with measurements near the longitudinal surface plasmon resonance of gold nanorods. This allows for real-time, in situ measurements of biomolecular layer thickness on nanoparticles with sub-nanometer precision.

Immediate Significance:

This work represents a methodological advance in nanoparticle characterization, providing a label-free tool for studying biomolecular interactions at nanoparticle surfaces. The technique enables real-time monitoring of surface modification processes and allows investigation of environmental effects such as temperature and ionic strength on surface coatings. As a complementary approach to existing characterization methods, it shows promise for applications in nanoparticle-based drug delivery systems.

Technical Suggestions:

1. The manuscript would benefit from a more detailed discussion of the technique's limitations, including minimum detectable layer thickness changes.

Thank you for your valuable feedback and insightful comments. In response, we have expanded the discussion at the end of the manuscript to provide a more comprehensive analysis of both the limitations and strengths of the technique.

2. The reversibility of temperature-dependent changes in CTAB layer (Figure 3) should be addressed.

Thank you for your suggestion. We have added a figure to the SI (Fig. S6) that demonstrates the ACFs returning to their original values after CTAB melting, indicating that the process is reversible.

3. The organization of Figure 4d feels disconnected from previous panels and needs better integration.

Thank you for your feedback. We have adjusted the dimensions of Fig. 4d to align with the other panels.

4. The fundamental principles behind depolarized forward scattering advantages (how separating rotational diffusion signals from translational Brownian motion) need clearer explanation for broader accessibility.

Thank you for highlighting this important point. We agree with your observation and acknowledge that this explanation was unintentionally omitted from the supplementary information in our initial submission. We have now included the relevant text in the supplementary materials and expanded it to provide a clearer and more accessible explanation of the fundamental principles behind depolarized forward scattering, including how it separates rotational diffusion signals from translational Brownian motion.

5. The authors should provide quantitative comparisons with existing methods to better highlight the specific improvements achieved.

In response to this suggestion, we have included a quantitative comparison with existing methods in the discussion section of the manuscript. This comparison highlights the specific improvements achieved by our technique, such as its sub-nanometer sensitivity, label-free operation, and ability to isolate rotational diffusion signals from translational motion. By providing these metrics, we aim to clearly demonstrate the advantages of our approach relative to other established methods. We hope this addition sufficiently addresses the comment.

6. The biological interaction studies (Figure 4) could be expanded to better demonstrate the method's practical advantages.

Thank you for your comment. While we appreciate the suggestion to expand the biological interaction studies in Figure 4, we respectfully disagree with the need for further expansion in this manuscript. The primary objective of this article is to demonstrate the broad capabilities of the technique, including its application to biomolecular interaction studies. A more comprehensive investigation of these interactions would require additional experiments and is beyond the scope of this work. Such an in-depth study would be more appropriately addressed in a dedicated future publication. We hope this clarifies our approach and the intended scope of this paper.

7. The manuscript structure should be reorganized to better emphasize practical applications rather than technical validation.

Thank you for your feedback. We would like to clarify that the primary purpose of this study is to provide a comprehensive technical validation of the method, demonstrating its applicability across various application scenarios. While practical applications are indeed important, the focus of this work is to establish the method's versatility and reliability, which lays the foundation for future studies exploring specific practical applications in greater depth. We believe this structure aligns with the goals of the study and the manuscript's intended contributions.

8. Claims about real time kinetic measurements should be supported with actual time-resolved studies.

We believe this concern may stem from a misunderstanding, as Figure 4B indeed presents time-resolved binding kinetics of two types of molecules to the surface of the nanorods, demonstrating the technique's capability for real-time kinetic measurements.

Reviewer 2

Johansson et al. present a novel method, so-called "depolarized forward light scattering (DFLS)" for determining fast rotational motion of a nanorod, most importantly its interaction with biomolecules with a subnanometer precision by demonstrating detection of temperature-induced dissociation of CTAB layer on gold nanorods. In addition, they studied adsorption kinetics of PEG and BSA at different ionic strengths. The work is very interesting. The proposed method is promising for applications of nanoparticles-biomolecules interactions. The paper can be published as it is now. However, the authors may consider some of my following minor comments before its publication.

(1) The viscosity (η) in equation 1 is only considered to be as a function of temperature η (T) as temperature change is due to the laser heating. However, I think that it is more complex than that. Viscosity will also be as a function of time as the particles rotates and so its absorption of linear polarization will change and so the temperature-rise. This means that η is as η (T, t). In addition, the laser induced heating is a gradient profile and so, η is η (T, t, r) where r is the distance from the particle surface. Can the authors comment on this?

We appreciate the reviewers' insightful comments regarding the role of temperature and viscosity variations in the surrounding water and their impact on rotational Brownian motion and diffusion. As discussed in, for example, the paper by Ruijgrok et al. (Phys. Rev. Lett. 107, 037401 (2011)), a heated particle is surrounded by a medium where the temperature and viscosity vary spatially. However, the Brownian motion and diffusion can be effectively described by an "effective temperature," which, at least for rotational Brownian motion, aligns closely with the particle temperature.

In our study, we anticipate that both the temperature and rotational diffusion constant will vary with the power absorbed by the particle. Nevertheless, as noted by Ruijgrok et al., the thermal equilibration time (approximately 10 ns) is significantly shorter than the characteristic timescale for rotational diffusion (hundreds of microseconds). This temporal separation supports our assumption that the diffusion constant depends on the particle's instantaneous orientation relative to the polarization direction of the incident light.

At a more detailed level, the autocorrelation function of the scattered light intensity is influenced by the rotational diffusion primarily through the decay of the angular momentum J=2 component in the orientational probability distribution function of the particle. Importantly, we observe that this has minimal impact on the autocorrelation function. Instead, the function behaves as if the diffusion constant is effectively set by an average temperature. For a deeper exploration of this aspect, we direct the reviewers to the supplementary material, where this issue is further addressed.

(2) For extinction spectroscopy, the authors used dichroic to separate the laser detection from white light. However, this blocks important information regarding the longitudinal plasmon. For nanoparticles aggregation or biomolecular interactions, extinction spectroscopy around longitudinal plasmon will be important. The authors can block only the narrow laser band with some filters and can detect the rest of the signal around the longitudinal plasmon. Can the authors comment on this?

We agree that the suggested approach would be an excellent way to retain valuable information about the longitudinal plasmon. We have noted this suggestion to improve the methodology in the text of the manuscript.

(3) Can the temperature-induced CTAB dissociation also be observed by extinction spectroscopy, specifically by longitudinal plasmon-shift?

Unfortunately, it is not possible to observe the temperature-induced CTAB dissociation through extinction spectroscopy. This is because the LSPR shift caused by CTAB dissociation is very small and is overshadowed by the bulk refractive index changes of the surrounding medium. These bulk changes induce a significant shift in the plasmon resonance, making it challenging to isolate and attribute any spectral changes specifically to the dissociation of CTAB. We have added this explanation to the manuscript text.

(4) Can the authors give some perspective in terms of in-vivo applications of DFLS method?

Thank you for this valuable question. We included the perspective in the final paragraphs of the manuscript: "The DFLS method holds significant potential for in vivo applications due to its ability to provide real-time, label-free measurements of molecular interactions and dynamics with high sensitivity. However, translating DFLS to in vivo applications requires further optimization, including improvements in signal-to-noise ratios, enhancements in penetration depth for measurements in tissue environments, and the integration of the method with optical setups tailored for biological imaging. With these technical challenges addressed in future developments, DFLS could become a powerful tool for probing molecular-scale interactions and characterizing the local environment within living organisms."
